# Supplementary material for: Alkyl Glycerol Ethers as Adaptogens
Source: Mar Drugs. 2022 Dec 21;21(1):4. doi: 10.3390/md21010004 (PMC9862039; doi:10.3390/md21010004)
Supplement: Supplementary file 1 [file marinedrugs-21-00004-s001.zip › marinedrugs-1948526-supplementary.pdf]

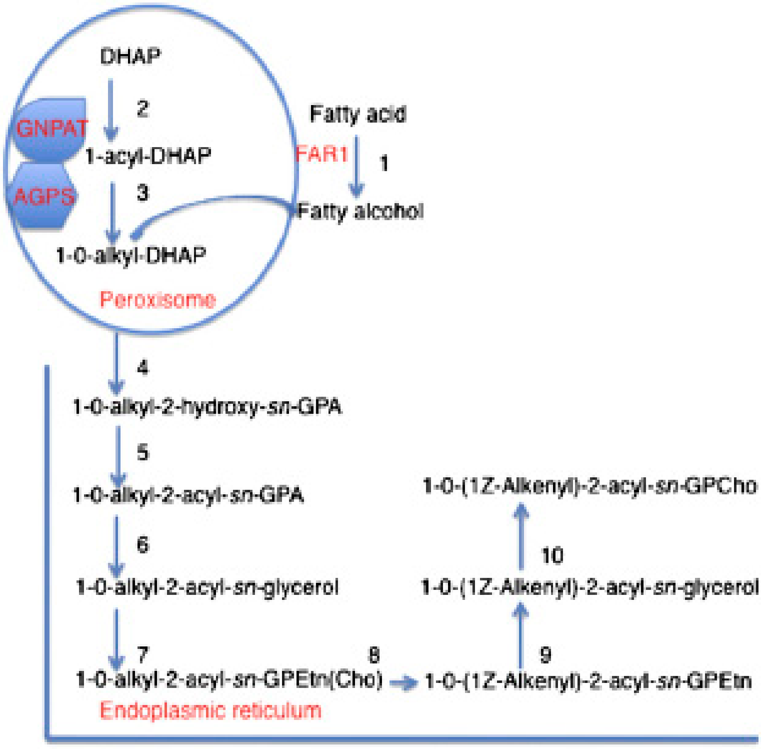

Figure S1. Metabolic pathway for plasmalogen synthesis.

Species indicated by (Cho) represent the choline equivalent of the corresponding GPEtn species. Enzymes are: (1) fatty alcoholreductase 1, (2) glycerone phosphate O-acyltransferase, (3) alkylglycerone phosphate synthase, (4) alkyl/acyl DHAP reductase, (5) alkyl/acyl glycerophosphate acyltransferase, (6) phosphatidic acid phosphatase, (7) ethanolamine (choline) phosphotransferase (8) plasmenylethanolamine desaturase, (9) phospholipase C, (10) choline phosphotransferase.

Braverman, N.E.; Moser, A.B. Functions of plasmalogen lipids in health and disease. *Biochimica et Biophysica Acta* **2012**, *1822*, 1442–1452. https://doi.org/10.1016/j.bbadis.2012.05.008
